# Structural modelling of wellbeing for Indigenous Australians: importance of mental health

**DOI:** 10.1186/s12913-019-4302-z

**Published:** 2019-07-15

**Authors:** Rosalie Schultz, Stephen Quinn, Byron Wilson, Tammy Abbott, Sheree Cairney

**Affiliations:** 1 Centre for Remote Health, Flinders University, PO Box 4066, Alice Springs, NT 0871 Australia; 2 Department of Statistics, Data Science and Epidemiology, Swinburne University, Melbourne, VIC Australia; 3 Menzies School of Health Research, Charles Darwin University, Darwin, NT Australia; 4 Ninti One Ltd, Cooperative Research Centre for Remote Economic Participation, Alice Springs, NT Australia

**Keywords:** Aboriginal Australians, Indigenous Australians, Functional health, Health care access, Mental health, Physical health, Remoteness, ARIA, Structural equation modelling, Wellbeing

## Abstract

**Background:**

Australia provides health care services for Indigenous peoples as part of its effort to enhance Indigenous peoples’ wellbeing. However, biomedical frameworks shape Australia’s health care system, often without reference to Indigenous wellbeing priorities.

Under Indigenous leadership the Interplay research project explored wellbeing for Indigenous Australians in remote regions, through defining and quantifying Indigenous people’s values and priorities. This article aimed to quantify relationships between health care access, mental and physical health, and wellbeing to guide services to enhance wellbeing for Indigenous Australians in remote regions.

**Methods:**

Indigenous and non-Indigenous researchers worked with Indigenous people in remote Australia to create a framework of wellbeing priorities. Indigenous community priorities were community, culture and empowerment; these interplay with government priorities for Indigenous development of health, education and employment.

The wellbeing framework was further explored in four Indigenous communities through a survey which measured aspects of the wellbeing priorities. Indigenous community researchers administered the survey in their home communities to 841 Indigenous people aged 15 to 34 years from June 2014.

From the survey items, exploratory factor analysis was used to develop constructs for mental and physical health, barriers to health care access and wellbeing. Relationships between these constructs were quantified through structural equation modelling.

**Results:**

Participants reported high levels of health and physical health (mean scores (3.17/4 [SD 0.96]; and 3.76/4 [SD 0.73]) and wellbeing 8.07/10 [SD 1.94]. Transport and costs comprised the construct for barriers to health care access (mean access score 0.89/1 [SD 0.28]).

Structural equation modelling showed that mental health, but not physical health was associated with wellbeing (β = 0.25, *P* < 0.001; β = − 0.038, *P* = 0.3). Health care access had an indirect positive relationship with wellbeing through mental health (β = 0.047, *P* = 0.007). Relationships differed significantly for participants in remote compared with those in very remote communities.

**Conclusions:**

Greater attention to mental health and recognition of the role of services outside the health care sector may have positive impacts on wellbeing for Indigenous people in remote/ very remote Australia. Aggregation of remote and very remote populations may obscure important differences between Indigenous communities.

## Background

Australia provides targeted health care and other services for Indigenous people with the aim of reducing their health and socio-economic disadvantage [1]. State, territory and federal Australian governments formally committed to reducing disparities between Indigenous and other Australians in 2008 through the Closing the Gap strategy [2]. Since then, progress has been limited: improvements in measures of Indigenous people’s health have stalled, and education and employment gaps are widening [3].

In Australia as globally, movements for Indigenous self-determination recognise that Indigenous communities have different goals and aspirations from those of non-Indigenous populations. Socio-economic indicators developed by and for national populations may not address Indigenous peoples’ aspirations [4]. Measuring and monitoring Indigenous community progress requires development of indicators that are meaningful for Indigenous people, and that address the distinct and diverse aspirations of individual communities. These can build on measurements of life satisfaction and wellbeing, which are fundamental to development, relatively simple to measure and monitor, and unbiased by differences in culture [4].

Recognising that Indigenous Australians have distinct social characteristics, the Australian Bureau of Statistics conducts a periodic National Aboriginal and Torres Strait Islander Social Survey (NATSISS); Aboriginal and Torres Strait Islander groups being the two populations of Indigenous Australians. The most recent NATSISS, conducted in 2014–2015, included a question on overall life-satisfaction. Results showed that overall, Indigenous Australians enjoy high levels of life satisfaction, with those in remote and very remote regions reporting mean life-satisfaction of 7.6, and those in non-remote regions 7.2 [5]. Mean life satisfaction score for all Australians in 2015 was also 7.6, despite Indigenous people in remote/ very remote regions showing significant differences from the mean Australian levels in other social measures including income, employment, education and health [6]. This suggests that at a population level, remote residence is associated with increased life satisfaction for Indigenous Australians.

**Fig. 1:**
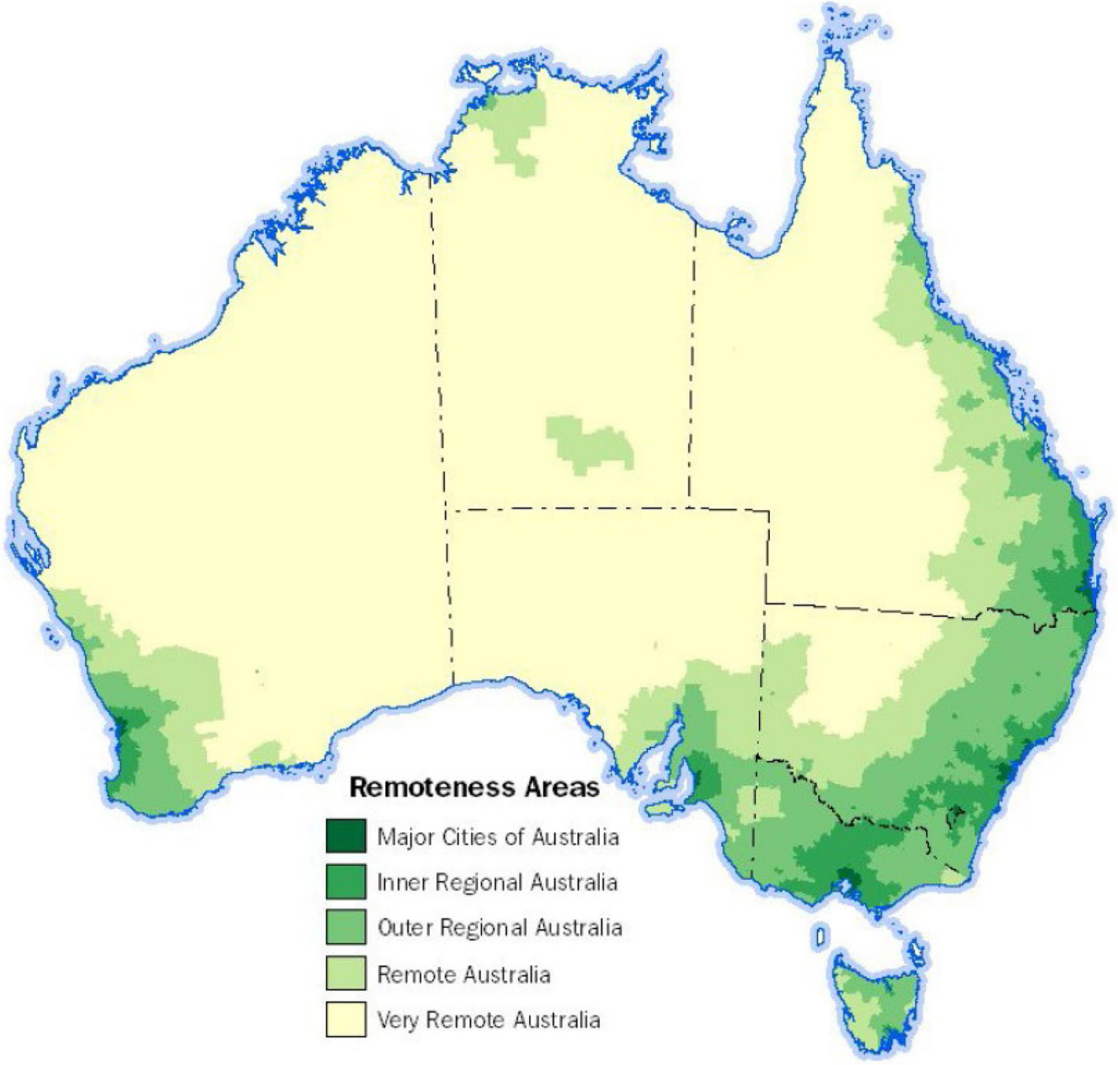
Map of the 2016 Remoteness Areas for Australia [7]

The Access and Remoteness Index of Australia (ARIA) defines five categories of remoteness based on road distance to the nearest urban centre, shown geographically in Fig. 1 [7]. The research described in this article involved communities in areas classified as remote and very remote. More Indigenous Australian live in very remote than remote regions, 95,200 and 53,500 respectively, 11.8 and 6.7% of the total Indigenous population [8].

There is a paradox regarding Indigenous people of remote and very remote Australia, who report high levels of wellbeing despite socio-economic disadvantage as assessed through indicators of education, employment and income [5, 9]. Possible explanations include the strength of identity and culture that Indigenous people maintain through their connections to the land particularly when they have access to their ancestral lands [10]. In other populations, improvements in wellbeing are independently associated with improvements in health, education and productivity, through increases in creativity, cognitive capacity, sociability, cooperation and productivity [11]. Health benefits of increases in wellbeing include reduced inflammation, lowered risk of cardiovascular disease and susceptibility to infections, and increases in health promoting behaviours including choosing healthier foods, exercising more and smoking less [11]. Although such benefits have not been specifically demonstrated for Indigenous Australians, attention to wellbeing may provide opportunities to address complex socio-economic disadvantage where current approaches are inadequate [3].

Wellbeing as a goal of service delivery was the focus of the Interplay research, an initiative of the Cooperative Research Centre for Remote Economic Participation whose overall aim was to guide economic development to meet the aspirations of Indigenous people in remote/ very remote regions of Australia [12, 13]. The Interplay research began by developing a wellbeing framework, which encompassed the government priorities of health, education and employment, together with community priorities of community, culture and empowerment [12]. Social and emotional wellbeing is often used as an alternative term for mental health for Indigenous Australians, but as the construct in this research related to absence of symptoms of mental illness, we use the term mental health [14].

The research presented here aims to quantify relationships between health care access, mental and physical health and wellbeing, assuming that access to health care services contributes to wellbeing both directly and through its impacts on mental and physical health. The hypothesis was that health care access is directly associated with wellbeing for Indigenous people in remote and very remote communities; alternatively, mental and physical health may mediate this relationship. Understanding these relationships can provide direction for services to optimise wellbeing for Indigenous Australians in remote/ very remote regions.

## Methods

### Research governance

The Cooperative Research Centre for Remote Economic Participation (CRC-REP) which has community connections throughout remote Australia managed the Interplay research [13]. Indigenous leadership and governance of the project prioritised qualitative approaches to understanding wellbeing, based on peoples’ stories as sources of knowledge and understanding. Government, university and industry partners in the project sought numerical indicators, so the project also included quantitative analysis of aspects of wellbeing.

Design and development of the research extended over 3 years of consultation with Indigenous communities and researchers. Advisory group meetings, workshops, interviews and discussions, and the employment of Indigenous community-based researchers enabled collaboration between Indigenous community members, researchers and government representatives in all aspects of the research [15]. Considerable effort was made to ensure that the project encompassed both Indigenous and non-Indigenous knowledge and understandings. Following data collection, the research team continues work with communities of the study. This keeps community researchers, participants and other community members informed of the analyses of the study and ensures support for wider reporting and implementation [16].

### Study population

Four communities in Northern Territory and Western Australia from the CRC-REP network identified themselves to participate in the research. To achieve broad representation of remote Indigenous communities, we included a range of geographies; population size and proportion Indigenous; and level of Indigenous language use [12]. Table 1 provides information on the communities involved in the study.

**Table 1:** Community geography, remoteness, language, and research participation

Community	1	2	3	4
Geography	River	Island	Desert	Coastal
Distance from major centre	300 km	500 km	1000 km	650 km
Remoteness classification	Remote	Very remote	Very remote	Very remote
Total population	9207	2550	1158	843
Proportion Indigenous	24.2%	88.6%	24.4%	75.3%
Primary community language	Kriol	Djambarrpuyngu	Martu	Gumatj
Proportion of Indigenous people who speak Indigenous languages at home	25.7%	98.1%	63.5%	84.3%

Population data from Australian Bureau of Statistics [17]

Communities of the study together with Indigenous service providers and leaders nationwide contributed to development of a wellbeing framework. The framework comprises Indigenous community priorities of community, culture and empowerment and government priorities of health, education and employment [18].

### Survey development and data collection

The Interplay wellbeing survey was developed to further explore wellbeing priorities. It included questions on demography, Indigenous status, mental and physical health symptoms and diagnoses, barriers to health care access, and wellbeing. As far as possible questions were developed from instruments which have been validated for Indigenous Australians. Experienced researchers worked closely with community researchers to ensure they shared understandings of the meaning of the survey questions, and that the questions could be translated to community languages if required [12]. The survey was designed to generate valid, reliable and quantifiable measures of contributors to Indigenous people’s wellbeing [12].

Through relationships and community networks, Indigenous Community Researchers recruited young adult participants to complete the survey in their home communities over 12 months from June 2014. The surveys were in English, but where necessary the community researchers who had been involved in the development of the survey used community languages to ensure that participants understood the meaning of the questions [12]. The community researchers administered surveys from iPads, taking approximately 1 hour per survey.

### Measures

The Interplay research used standard measurement tools as far as possible. To measure physical health, questions involved health as a resource for living, through asking people whether health problems interfered with aspects of their day to day lives [12]. Refined questions from the Strong Souls instrument provided a measurement of mental health [14]. The absence of specific barriers to seeking health care formed the construct for health care access, while current life satisfaction was the measurement of wellbeing [19]. Education and employment measures were completed years of school and employment status respectively, based on questions from the Australian census [17]. Remoteness was determined by the ARIA classification of the community where the participant completed the survey [7].

### Statistical analysis

Structural equation modelling enabled factors from the Interplay wellbeing framework to be developed into measurable constructs, to analyse, interpret and report in meaningful ways to both Indigenous and non-Indigenous communities [12]. Data analysis was conducted using SPSS Statistics Software version 24 and AMOS version 23 [20]. Missing data were estimated using multiple imputations taking the median as the most likely value. Exploratory factor analysis (EFA) was used to develop constructs for mental and physical health and health care access from the survey items, using maximal likelihood extraction with promax rotation [21]. Three constructs for health care access and mental and physical health had strong factor loadings (> 0.4), no items with cross-loadings, discriminant and face validity and adequate reliability.

We tested hypothesised relationships between health care access and mental and physical health and wellbeing through confirmatory factor analysis (CFA) using structural equation modelling in AMOS. Bootstrapping enabled mediation analysis to further assess relationships between constructs [22]. Model fit was assessed using a range of types of fit indices, namely χ^2^ to degrees of freedom ratio, non-normed fit index (NNFI), comparative fit index (CFI), Akaike’s Information Criteria (AIC) closer to saturated model than the independence model and Root Mean Square Error of Approximation (RMSEA) with confidence interval [23].

While participant numbers were not large enough to analyse differences between the communities in the research, multigroup analysis was performed to explore differences between participants in very remote and those in remote communities [7].

## Results

### Participant demography

Across the four communities of the study, 841 Indigenous participants completed surveys. Mean age was 25.2 years, SD = 5.34, range 15 to 34 years. Females made up 489 (58.1%) of respondents. Based on 2011 census population which was the nearest to the date of the research, participants made up 45% of Indigenous people in the target age group in the study communities [17]. Participants’ community, education and employment status, and relationships of these variables with wellbeing are shown in Table 2.

**Table 2:** Number and percentage of participants by community, education level and employment status reporting levels of wellbeing

Demographic variable	Community number and remoteness	Wellbeing level			
		Low (0 to 4)	Moderate (5 to 7)	High (8 to 10)	Total
Community	1 Remote	21	174	350	545
		3.9%	31.9%	64.2%	100%
	2 Very remote	1	24	26	51
		2.0%	47.1%	51.0%	100%
	3 Very remote	2	28	74	104
		1.9%	26.9%	71.2%	100%
	4 Very remote	4	54	83	141
		2.8%	38.3%	58.9%	100%
Highest level of schooling	Primary school	4	29	21	65
		6.2%	44.6%	41.2%	100%
	Junior high school (years 8 to 10)	21	204	367	592
		3.5%	34.5%	62.0%	100%
	Senior high school (years 11 to 12)	3	47*	134*	184
		1.6%	25.5%	72.8%	100%
Employment status	No paid employment	14	146	251	411
		3.4%	35.5%	61.1%	100%
	Part time employment	8	79	160	247
		3.2%	32.0%	64.8%	100%
	Full time employment	6	55	122	183
		3.3%	30.1%	66.7%	100%
Total		28	280	533	841
		3.3%	33.3%	63.3%	100%

*Indicates value is different from expected based on *P* < 0.05

Relationships of demographic variables with wellbeing

Education: χ^2^ = 14.1, df = 4, *P* = 0.007

Employment: χ^2^ = 1.07, df = 4, *P* = 0.72

Community: χ^2^ = 9.74, df = 6, *P* = 0.14

### Descriptive statistics

Survey participants described good physical health, with over 88% reporting no interference from health problems with their normal daily activities, energy levels, socialising, or work/ study. However, symptoms of depression and anxiety were common, with nearly half the respondents reporting at least one depression or anxiety symptom. The main barriers to accessing health care were transport (14.0%), cost (8.4%), cultural and language concerns (7.1%) and privacy (4.9%). Participants reported high levels of wellbeing, with mean score 8.1/10 (SD 1.94). Means and standard deviations are summarised in Table 3.

**Table 3:** Survey questions on mental and physical health, access to health care and wellbeing with mean response and standard deviation

Construct	Survey questions	Mean	SD
Mental health: Have you felt any of these from too many worries in the last few weeks? (0–4, with high scores indicating good health)	Hard to breathe	3.41	1.07
	Dizzy	3.32	1.13
	Shaky	3.42	1.05
	Get angry or wild real quick	2.87	1.33
	Too many bad moods	2.94	1.28
	Trouble sleeping	3.09	1.33
Physical health: Have health problems got in the way of these in the last few weeks? (0–4, with high scores indicating good health)	Normal activities	3.77	0.77
	Work or study	3.85	0.63
	Energy levels	3.75	0.84
	Socialising with family or friends	3.67	0.99
Health care access: Do any of these things make it hard to use health care? (0–1, with 1 being no barrier)	Costs/ money	0.92	0.28
	Transport	0.86	0.35
	Culture/ language	0.93	0.26
	Privacy	0.95	0.21
Wellbeing:	On a scale of 1 to 10 how well is your life going?	8.07	1.94

*n* = 841

#### Exploratory factor analysis

We used maximal likelihood extraction to identify constructs for physical and mental health, and health care access.

Capacity for normal daily activities, work/ study, socialising, and energy levels formed the construct for physical health. There was a high degree of reliability of these factors for the physical health construct (Cronbach alpha reliability 0.92), and participants had mean physical health score of 3.76/4 (SD 0.96).

Symptoms of anxiety (feeling dizzy, feeling shaky, and hard to breathe), and depression (bad moods, quick to anger and difficulty sleeping) formed the construct of mental health. The items showed a high level of reliability (Cronbach alpha reliability 0.88). Mean mental health score was 3.17/4 (SD 0.96).

Costs and transport made up the construct for barriers to health care access, with Cronbach alpha reliability 0.74. Cultural and language barriers and privacy did not load strongly onto the construct (0.42, and 0.28) so the final model did not include these factors.

Distributions and correlations of the constructs in the model are shown in Table 4.

**Table 4:** Constructs of mental health, physical health, health care access, with wellbeing and variate correlations

Construct	Range	Mean	SD	Cronbach α reliability	Skewness	Kurtosis	Bivariate correlations		
							Mental health	Physical health	Health care access
Mental health	0–4	3.17	0.96	0.88	−1.29	1.19			
Physical health	0–4	3.76	0.73	0.92	−3.26	10.21	0.16 ***		
Health care access	0–1	0.89	0.28	0.74	−2.40	4.41	0.23***	0.25***	
Wellbeing	1–10	8.07	1.94	Single item	−0.65	−0.63	0.24***	−0.039 NS	−0.088*

*n* = 841

*SD* standard deviation

***P < 0.001, *P < 0.05, *NS* not significant

### Model development and confirmatory factor analysis

**Fig. 2:**
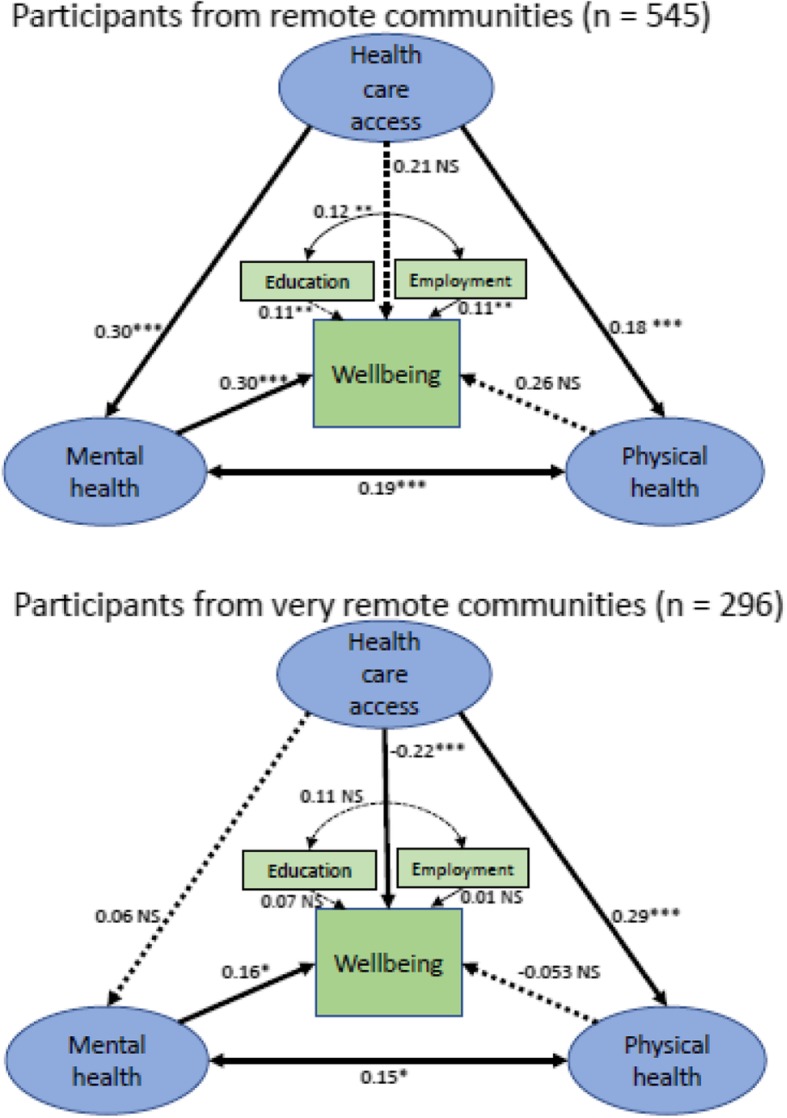
Structural equation models showing relationships between health care access, and mental and physical health to wellbeing for participants in remote and very remote communities. ***P < 0.001; **P < 0.01; *P < 0.05; NS = not statistically significant. Model fit indices: χ^2^ = 345.13; df = 158; χ^2^/df = 2.18. NNFI = 0.96, CFI = 0.97, Model AIC = 509, Saturated AIC = 480, Independence AIC = 6548; RMSEA = 0.038, 90% confidence interval [0.032, 0.043]. Non-significant pathways contributing to the hypotheses are included as dotted lines in the diagrams

We developed a structural model to quantify relationships between mental and physical health, health care access and wellbeing. Education and employment were considered as covariates, and participants were grouped by the remoteness of their community. We anticipated negative skew and positive kurtosis of the constructs from the descriptive statistics, but the data fitted the model as shown in Fig. 2 with participants grouped into remote and very remote communities.

Health care access was not statistically associated with wellbeing for participants from remote communities, and had a negative association with wellbeing for participants from very remote communities (for participants in remote communities, β = 0.21, 95% confidence interval [− 0.087, 0.14], *P* = 0.6; for participants in very remote communities β = − 0.22, 95% confidence interval [− 0.35, − 0.076], *P* = 0.001). Mental health was associated with wellbeing for both groups (remote β = 0.30, [0.18, 0.41], *P* < 0.001; very remote β = 0.16, [0.053, 0.27], *P* = 0.014) while physical health was not statistically significantly associated with wellbeing for either group (remote β = 0.026, [− 0.08, 0.12], *P* = 0.5; very remote β = − 0.053, [− 0.15, 0.056] *P* = 0.4). Health care access was positively associated with mental health for participants from remote but not very remote communities (remote β = 0.30, [0.15, 0.46], *P* < 0.001; very remote β = 0.06, [− 0.072, 0.22], *P* = 0.4). Table 5 shows standardised regression weights, 95% confidence intervals and *P* values for direct relationships with wellbeing in the model.

**Table 5:** Standardised regression weights for constructs and wellbeing for participants from remote and very remote communities

	Relationship with wellbeing for participants by community		
	Remote community participants	Very remote community participant	All participants
Health care access	0.21 [−0.087, 0.14] *P* = 0.6	−0.22** [− 0.35, − 0.076] *P* = 0.001	−0.077 [− 0.17, 0.004] *P* = 0.05
Mental health	0.30*** [0.18, 0.41] *P* < 0.001	0.16* [0.053, 0.27] *P* = 0.014	0.25*** [0.17, 0.32] *P* < 0.001
Physical health	0.026 [− 0.08, 0.12] *P* = 0.5	− 0.053 [− 0.15, 0.056] *P* = 0.4	−0.038 [− 0.11, 0.041] *P* = 0.3

Relationship, 95% confidence interval, *P* value

***P < 0.001; **P < 0.01; *P < 0.05; *NS* not statistically significant

We explored the relationship between health care access and wellbeing further through mediation analysis. Physical health had non-significant relationships with wellbeing so was not further considered, while mental health was positively associated with both health care access and wellbeing for remote communities, so was potentially a mediating variable for health care access on wellbeing in remote but not very remote communities.

We found a statistically significant positive indirect effect of health care access through mental health on wellbeing for participants from remote communities. There was also a positive indirect relationship of health care access on wellbeing, through mental health for all participants. The total relationship of health care access with wellbeing was positive for those in remote communities (Total relationship = 0.12, 95% confidence interval [0.036, 0.21], *P* = 0.005); negative for those in very remote communities (Total relationship = − 0.23, 95% confidence interval [− 0.34, − 0.082], *P* = 0.005) and not statistically significant when all participants were considered together (Total relationship = − 0.03, 95% confidence interval [− 0.12, 0.05], *P* = 0.5). These data are shown in Table 6.

**Table 6:** Direct, indirect and total relationships between health care access and wellbeing, for remote and very remote community participants

Health care access relationship to wellbeing	Remote community participants	Very remote community participants	All participants
Direct relationship	0.21 [−0.087, 0.14] *P* = 0.6	−0.22** [− 0.35, − 0.076] *P* = 0.001	−0.077 [− 0.17, 0.004] *P* = 0.05
Indirect relationship through mental health	0.11** [0.043, 0.17] *P* = 0.002	−0.006 [− 0.055, 0.029] *P* = 0.7	0.047** [0.015, 0.087] *P* = 0.007
Total relationship	0.12** [0.036, 0.21] *P* = 0.005	−0.23** [− 0.34, − 0.082] *P* = 0.005	−0.03 [− 0.12, 0.05] *P* = 0.5

Relationship, 95% confidence interval, *P* value

***P < 0.001; **P < 0.01; *P < 0.05; *NS* not statistically significant

## Discussion

### Interplay between health care access, mental and physical health and wellbeing

The hypothesis that health care access is associated with wellbeing was not confirmed. The relationship was not statistically significant for participants in remote communities and was negative for participants in very remote communities, signifying that greater health care access was associated with lower levels of wellbeing for participants in very remote communities. Mediation analysis showed an indirect positive effect of health care access on wellbeing through mental health for participants in remote communities, and this contributed to a positive total effect. For participants in very remote communities, the indirect effect was not significant, and the total effect of health care access on wellbeing remained negative. Thus, health care access does not have a positive relationship with wellbeing, and relationships between health care access and wellbeing differ for participants in remote and very remote communities.

Mental health was positively associated with wellbeing for participants in both remote and very remote communities. For those in remote communities, this indirect effect contributed to a positive overall effect of health care access on wellbeing.

Relationships between health care access and wellbeing are complex. Interactions between Indigenous people and health care providers do not consistently contribute to wellbeing [24, 25]. In Australia, health care in very remote regions is usually provided by non-Indigenous practitioners even where Indigenous people are the majority of the population. Staff turnover is high, this and can reinforce difference and negative attitudes between health care providers and Indigenous people [26, 27]. Different priorities and poor communication between Indigenous people and health care providers can contribute to experiences of disempowerment and alienation, and may undermine improvements in wellbeing that access to health care could provide [26, 28]. While efforts are made to overcome these issues, greater attention to mental health and to services outside the health sector may contribute to wellbeing for Indigenous people, especially in very remote communities [28].

### Remoteness

Demographic and socio-economic descriptions of Australians often aggregate remote and very remote populations who together they make up only 1.5% of the Australian people [29]. The separation between remote/ very remote and non-remote populations is also used for Indigenous Australians [9]. However, the concept of remoteness does not exist for many Indigenous Australians, and more Indigenous people live in very remote regions than remote regions. This contrasts with non-Indigenous Australians whose population declines with increasing remoteness [8]. The model presented here suggests that for Indigenous Australians the remote/ very remote aggregation may overlook important differences.

### Wellbeing

The high level of wellbeing 8.1/10 reported by Indigenous people in this study is consistent with other data such as the NATSISS [5] which indicates that Indigenous people in remote / very remote regions enjoy greater wellbeing than those in urban regions. There is little in the literature that explores the high levels of wellbeing of Indigenous people of remote/ very remote regions [30]. Instead most research focusses on negative indicators of Indigenous people in remote/ very remote Australia, including disease rates, life expectancy, unemployment, school attendance, literacy and numeracy [3].

Participants in this study also reported experiencing high levels of functional health, despite the high burden of disease of Indigenous people in remote Australia [9]. Mental health symptoms were more common than physical health problems, which may reflect the high burden of suffering among Indigenous communities attributed to stress, racism, and on-going oppression [31]. However the model suggests that recognising and managing the burden of mental health symptoms provides an opportunity for health care providers to significantly enhance wellbeing for people in remote regions [12].

### Barriers to health care access

Transport and costs were the factors that comprised the construct of health care access. These barriers to health care access have been identified for Indigenous people in settings across Australia [32–34]. Cultural and language differences were identified as barriers to health care access in the descriptive data in this project, and have been identified as important barriers for Indigenous Australians in other research [2]. However, they had low loadings in exploratory factor analysis and reduced the statistical fit of the model. Privacy was also identified in descriptive data as a barrier to health care access but did not load strongly onto the construct of health care access.

### Health and wellbeing for indigenous people

Australian Indigenous people’s understandings of health and wellbeing were defined in the 1989 National Aboriginal Health Strategy (NAHS) and remain in the current National Aboriginal and Torres Strait Islander National health plan 2013 to 2023 [2, 35].

“Health to Aboriginal peoples is a matter of determining all aspects of their life, including control over their physical environment, of dignity, of community self-esteem, and of justice. It is not merely a matter of the provision of doctors, hospital, medicine or the absence of disease and incapacity.” [35] page ix.

“[Health is] not just the physical well-being of the individual but the social, emotional, and cultural well-being of the whole community.” [35] page x

The Interplay project identified health as one of six wellbeing priorities, together with community, culture, and empowerment, education and employment. In the model here, mental health, which is freedom from depression and anxiety symptoms, was associated with wellbeing for participants in both remote and very remote regions, and mediated the relationship between health care access and wellbeing for participants in remote regions. Promoting mental health may be an important strategy for enhancing wellbeing for Indigenous people in remote and very remote Australia.

### Implications for service provision

The Interplay project provides an integrated framework to understand Indigenous wellbeing, and guide development of effective services. Since mental health is associated with wellbeing, services that contribute to mental health may enhance wellbeing more effectively than health care directed to physical health. This highlights the importance of services outside the health sector to wellbeing, which contribute to comprehensive primary health care, originally conceptualised as an intersectoral undertaking [36].

Services outside the health sector contribute to social and emotional wellbeing, which arise from all aspects of Indigenous people’s lives rather than being limited to aspects of health [10, 37]. Services aimed to enhance the strengths of Indigenous people and communities, including commitment to interpersonal relationships, cultural knowledge and language may contribute to the transformative approach required to reduced health and socio-economic disadvantage of Indigenous people [12]. Health services based on caring for ancestral lands, the basis of Indigenous health, rather than clinical imperatives may contribute to improved health and wellbeing outcomes [38].

Within the health sector, there is widespread recognition that interventions that effectively address mental health of Indigenous Australians will improve people’s wellbeing [39]. Key elements of interventions likely to be effective include delivery outside clinical spaces; attention to the specific needs of Indigenous peoples including historical policies of removing people from their families; and focus on empowerment and self-determination [39]. Ensuring that Indigenous people maintain control of services to address their mental health needs and that interventions are rigorously evaluated would contribute to improving mental health outcomes [39].

Primary health care for Indigenous Australians is increasingly driven by performance indicators related to physical health, rather than community needs and aspirations [40]. As part of the closing the gap strategy of reducing the disadvantage of Indigenous Australians, a set of numerical indicators of physical health, such as blood pressure, blood sugar and body weight has been defined. Health services are required to report these to government funding agencies annually [41]. How this intense monitoring affects people’s wellbeing or health care access has not been considered. The rationale is to drive services to closely monitor people’s clinical status and behaviour through the performance of health care services [41]. While it is conceivable that improvement in physical health indicators may contribute to mental health, interventions specifically established to improve mental health for Indigenous people may be more effective [42]. Improvements in mental health and wellbeing may then lead to improved physical health, as suggested in the Interplay structural model and in the literature [43]. Mental health complements other contributors to wellbeing identified in the Interplay project, namely cultural practices, empowerment, identity and spirituality, Indigenous and English literacy, employment, community and freedom from substance use [12].

Australia’s Indigenous community-controlled health sector has long-advocated for a broader approach to health but been limited by funding requirements that demand a focus on biomedical services [40]. This limits both the impact of health care on wellbeing, and also the impact of health care on health because the very meaning of health for Indigenous people may not be represented in the biomedical model [12]. Effective community-control of Indigenous health care services and better integration of services may have manifold benefits, through a comprehensive approach including action on the social determinants of health, and through greater levels of employment of Indigenous people [26].

### Study limitations

Limitations of this study include its localised scale, providing detailed information about a convenience sample of participants from four Indigenous communities in remote/ very remote regions rather than a statistically representative sample. Data are cross-sectional so direction of relationships is theoretical rather than experimental. Surveys were conducted by community researchers in their home communities so interpersonal relationships may have led to response bias.

While the survey instrument was developed by experienced researchers working with community researchers, and there was agreement about the meaning of the questions, the accuracy and consistency of interpretations have not been formally established.

Owing to small numbers of participants from individual communities, sample size was inadequate to conduct multigroup analysis by community [44]. The analysis with participants grouped by community remoteness highlights the possible differences between communities, and suggests that this may be an important area of further research.

Lack of clinical data and more specific measures of health care access are limitations. Relationships between health care access, biomedical measures of health and Indigenous people’s own experiences of health and wellbeing form an important area for further study [26, 45].

## Conclusions

The Interplay project worked with Indigenous people in remote/ very remote regions of Australia to explore wellbeing, which is an outcome of service provision. Structural equation modelling of wellbeing and its relationships with health care access and mental and physical health showed that of these constructs, only mental health is associated with wellbeing. For participants in remote communities, mental health also forms an indirect pathway from health care access to wellbeing. Relationships differed between participants from remote and very remote communities.

Mental health and wellbeing for Indigenous Australians in remote Australia may be enhanced through strengthening and collaboration among services outside the health sector, particularly those that contribute to relationships, empowerment, cultural identity and care of the land. Addressing wellbeing may contribute to alleviation of other aspects of socioeconomic disadvantage faced by Indigenous Australians.

## Data Availability

The survey instrument and data that support the findings of this study are held by Ninti One Incorporated. Restrictions apply to their availability, as both were used under license for the current study, and so are not publicly available. Survey Instrument and data are however available from the authors upon reasonable request and with permission of Ninti One.

## Abbreviations

ACI: Akaike’s Information Criteria
AGFI: Adjusted Goodness of Fit Index
CFA: Confirmatory factor analysis
CFI: Comparative Fit Index
CRC-REP: Cooperative Research Centre for Remote Economic Participation
EFA: Exploratory factor analysis
GFI: Goodness of Fit Index
NATSISS: National Aboriginal and Torres Strait Islander Social Survey
NNFI: Non-Normed Fit Index
RMSEA: Root Mean Square Error of Approximation
SD: Standard deviation
SEM: Structural Equation Modelling
